# Phylogenetically Diverse Aerobic Anoxygenic Phototrophic Bacteria Isolated from Epilithic Biofilms in Tama River, Japan

**DOI:** 10.1264/jsme2.ME15209

**Published:** 2016-07-23

**Authors:** Setsuko Hirose, Katsumi Matsuura, Shin Haruta

**Affiliations:** 1 Department of Biological Sciences, Tokyo Metropolitan University1–1 Minami-Osawa, Hachiojii, TokyoJapan, 192–0397

**Keywords:** aerobic anoxygenic phototrophic bacteria, river epilithic biofilm, isolation, phylogenetic diversity, novel species

## Abstract

The diversity of aerobic anoxygenic phototrophic (AAP) bacteria in freshwater environments, particularly in rivers, has not been examined in as much detail as in ocean environments. In the present study, we investigated the phylogenetic and physiological diversities of AAP bacteria in biofilms that developed on submerged stones in a freshwater river using culture methods. The biofilms collected were homogenized and inoculated on solid media and incubated aerobically in the dark. Sixty-eight red-, pink-, yellow-, orange-, or brown-colored colonies were isolated, and, of these, 28 isolates contained the photosynthetic pigment, bacteriochlorophyll (BChl) *a*. Phylogenetic analyses based on 16S rRNA gene sequences showed that the isolates were classified into 14 groups in 8 operational taxonomic units (OTUs) and distributed in the orders *Rhodospirillales*, *Rhodobacterales*, and *Sphingomonadales* of *Alphaproteobacteria* and in *Betaproteobacteria*. Physiological analyses confirmed that none of the representative isolates from any of the groups grew under anaerobic phototrophic conditions. Seven isolates in 4 OTUs showed a 16S rRNA gene sequence identity of 98.0% or less with any established species, suggesting the presence of previously undescribed species of AAP bacteria. Six isolates in 2 other OTUs had the closest relatives, which have not been reported to be AAP bacteria. Physiological comparisons among the isolates revealed differences in preferences for nutrient concentrations, BChl contents, and light-harvesting proteins. These results suggest that diverse and previously unknown AAP bacteria inhabit river biofilms.

Aerobic anoxygenic phototrophic (AAP) bacteria grow heterotrophically by aerobic respiration. They produce bacteriochlorophyll (BChl), but do not grow anaerobically in the light (39). Imhoff and Hiraishi (16) suggested that AAP bacteria use photosynthesis as a supplementary energy source.

More than 80 species of AAP bacteria have been described since their discovery in 1978 (11, 38). Although these bacteria mainly belong to the class *Alphaproteobacteria*, a few species have also been found in the classes *Betaproteobacteria* and *Gammaproteobacteria* (55). Novel phototrophic species were recently identified in the phyla *Acidobacteria* and *Gemmatimonadetes* (45, 59). These species are metabolically similar to AAP bacteria. AAP bacteria are widespread in the ocean, representing a considerable fraction, up to 24%, of the marine bacterioplankton, and play significant roles in marine carbon cycling (23–25). However, the distribution and diversity of AAP bacteria in freshwater environments are still poorly understood.

The presence of anoxygenic phototrophic bacteria in freshwater lakes and rivers has been detected using functional genes, such as the anoxygenic photosynthesis gene *pufM*, which encodes for the small subunit of the photochemical reaction center (4, 18, 37, 49, 57), and infra-red fluorescence (4, 8, 29). We also reported the diversity of *pufM* gene sequences in river epilithic biofilms (15), which appear to be adequate habitats for AAP bacteria because oxygen, organic nutrients, and light are available. These findings indicate the high phylogenetic diversity of anoxygenic phototrophic bacteria in freshwater environments and the presence of novel AAP bacteria. However, most of these bacteria have yet to be isolated and their characteristics currently remain unclear. Isolation and cultivation analyses are necessary for identifying and characterizing AAP bacteria. To the best of our knowledge, an AAP cultivation study on river biofilms has not yet been performed.

In the present study, we isolated BChl *a*-containing bacteria from epilithic biofilms in the upper reaches of Tama River, Japan and characterized them phylogenetically and physiologically as AAP bacteria.

## Materials and Methods

### Sampling of epilithic biofilms

Three submerged cobbles with a maximum length of approximately 15 to 20 cm were collected from within a 10-m^2^ area of a streambed in a sunny location at the upper reaches of Tama River in Ome City (35°47′13″N, 139°15′15″E, altitude of 200 m), Tokyo, Japan on February 20 and July 23, 2012. Biofilms with thicknesses of approximately 0.5–1 mm were present on the cobbles. A 150-cm^2^ area of a diatom-dominated epilithic biofilm was scraped off of the top surface of each cobble using a sterilized toothbrush and suspended in 10 mL of sterilized distilled water. The suspensions were kept cool in ice and brought to the laboratory.

The river width and water depth at the sampling site were approximately 40 m and 0.2 m, respectively. The water temperature, pH, and flow velocity of river water at the sampling time were 3.0°C, 8.0, and 0.6 to 0.9 m s^−1^ in February and 13.8°C, 8.2, and 0.3 to 1.2 m s^−1^ in July. Average values for biochemical oxygen demand (BOD), dissolved oxygen (DO), total nitrogen (TN), and total phosphorus (TP) in river water in this region were 0.26±0.28 mg L^−1^ BOD, 11.8±0.7 mg L^−1^ DO, 0.58±0.05 mg L^−1^ TN, and 0.013±0.014 mg L^−1^ TP in February 2012 and 0.73±0.17 mg L^−1^ BOD, 9.6±0.2 mg L^−1^ DO, 0.75±0.04 mg L^−1^ TN, and 0.009±0.003 mg L^−1^ TP in July 2012 (monthly report by the Bureau of Environment, Tokyo Metropolitan Government, http://www.kankyo.metro.tokyo.jp).

### Isolation of BChl *a*-containing aerobic bacteria

Biofilms were dispersed using POLYTRON PT10/35 (KINEMATICA, Luzern, Switzerland) in suspensions in test tubes on ice. Suspensions were then serially diluted and spread onto agar plates. Gellan gum was also used instead of agar. Isolation was conducted using different dilutions of 2 types of media, PE medium (10) and Nutrient agar (NA, Eiken Chemical, Tochigi, Japan). PE medium (pH 7.5) contained (L^−1^) 0.5 g of sodium glutamate, 0.5 g of sodium succinate, 0.5 g of sodium acetate, 0.5 g of yeast extract (Wako, Osaka, Japan), 0.5 g of casamino acids, 1 mL of a vitamin mixture (10), and 5 mL of a basal salt solution (10). 1/5 PE medium is simply PE medium diluted 5 times. 1/10 PE medium or 1/100 PE medium contained a 10-fold or 100-fold lower concentration of sodium glutamate, sodium succinate, sodium acetate, yeast extract, casamino acids, sodium thiosulfate, and (NH_4_)_2_SO_4_. 1/10 and 1/100 PE medium both contained the same concentrations of KH_2_PO_4_, K_2_HPO_4_, basal salt solution, and 1/5 the concentration of the vitamin mixture as PE medium and the pH of media were adjusted to 7.5. Agar (1.5%, w/v) or gellan gum (2%, w/v) supplemented PE medium were designated as PEA or PEG, respectively. NA diluted 6 or 60 times (1/6 NA or 1/60 NA) was also used, supplied with agar up to 1.5%. The pH of diluted NA was adjusted to 7.0. Inoculated plates were cultivated aerobically at 30°C in the dark for 14 d.

After cultivation, randomly selected red-, pink-, yellow-, orange-, or brown-colored colonies were picked up. Absorption spectra of the colony suspension in distilled water were measured using a Jasco model V-630 spectrophotometer (Jasco, Tokyo, Japan). Colonies that showed absorbance maxima between 800 nm and 875 nm were selected, transferred to fresh 1/10 PEA plates, and cultivated aerobically at 30°C in the dark for 1 week. Transfer and cultivation were repeated more than twice in order to obtain pure cultures. Winter isolates (February) were indicated by the ‘W’ prefix, for example W09, and summer isolates (July) by the ‘S’ prefix.

### Growth properties

The growth properties of the isolates were examined under the following conditions. Anaerobic light conditions; test tubes were sealed with butyl rubber caps and the gas phase of each tube was substituted with nitrogen gas. Inoculated tubes were incubated under filtered incandescent light (*ca*. 2,000 lux) with a wavelength greater than 700 nm. Aerobic dark conditions; test tubes were equipped with the silicon plug ‘SILICOSEN’ (Shin-etsu Polymer, Tokyo, Japan) to let air through, set in a shaker at an angle, and shaken at 140 rpm in darkness. All cultivations were conducted at 30°C, using test tubes that were 18 mm in diameter and 180 mm in length containing 10 mL of liquid medium after the inoculation of 0.1 mL of the exponential phase of growth or early stationary phase in 1/5 PE medium under aerobic dark conditions. Growth was monitored at an optical density of 660 nm (mini photo 518R, Taitec, Saitama, Japan). Tests were performed with 5 replicates.

### BChl contents

Isolates were cultivated under aerobic dark conditions in 1/5 PE medium or PE medium and cells were harvested at the exponential phase of growth or early stationary phase and washed twice with 10 mM Tris-HCl buffer pH 8.0. BChl was extracted in acetone:methanol (7:2, v/v) from cells and absorbance at 770 nm was measured using a Shimadzu model UV-1800 UV-VIS spectrophotometer (Shimadzu, Kyoto, Japan). A millimolar extinction coefficient of 75 cm^−1^ was used to assess BChl contents (3). Dry cell weight was measured with harvested cells, washed twice with 10 mM Tris-HCl buffer pH 8.0 and dried at 80°C for 3 d.

### Rhodobacter sphaeroides

ATCC 17023^T^ (=2.4.1^T^), a reference strain obtained from the American Type Culture Collection was cultivated in 1/5 PE medium or PE medium under aerobic dark or anaerobic light conditions.

### Absorption spectra of disrupted cells

In order to avoid cell scattering, cells were disrupted before the measurement of *in vivo* absorption spectra. Cultures were grown aerobically in the dark for 7 d in 500-mL Sakaguchi flasks containing 100 mL of 1/5 PE medium on a reciprocal shaker. Cells were harvested and suspended in 10 mM Tris-HCl pH 8.0, disrupted by sonication at 130 W for 4 min in a test tube on ice (Sonicator, Ohtake Works, Tokyo, Japan), and centrifuged at 8,700×*g* for 4 min to remove cell debris. The supernatant was used for absorption spectra measurements using a Shimadzu model UV-1800 UV-VIS spectrophotometer (Shimadzu).

### Sequencing of the 16S rRNA gene and phylogenetic analyses

The total genomic DNA of the isolates was extracted according to Noll *et al.* (32). DNA fragments of the partial 16S rRNA gene were amplified using the primer sets 27F/1492R (26, 43). DNA sequences were obtained with the BigDye v3.1 cycle sequencing kit (Applied Biosystems, Foster City, CA, USA) and DNA sequencer ABI3130xl (Applied Biosystems) using the sequence primers 27F, 515F, 968F, 517R, 907R, and 1492R (30, 47, 50). Sequences were assembled into contigs using the ATGC program (GENETYX Ver. 12, GENETYX, Tokyo, Japan). Phylogenetic relatives were determined based on 16S rRNA gene sequences using a BLAST search (http://blast.ncbi.nlm.nih.gov/Blast.cgi). The ClustalW program was used for alignment and phylogenetic analyses were conducted with the neighbor-joining and maximum-likelihood algorithm using the MEGA version 6 program (44).

### Nucleotide sequence accession numbers

The partial bacterial 16S rRNA gene sequences obtained in this study have been deposited in the GenBank/EMBL/DDBJ database under accession numbers LC094480 to LC094493.

## Results

### BChl-containing colonies from epilithic biofilms

Table 1 summarizes the isolation results. Between 2.0×10^4^ and 1.5×10^5^ CFU cm^−2^ were obtained from the biofilms used in this study. The number of colonies did not vary widely among media. Many colonies were red, pink, yellow, orange, or brown in color. The absorption spectrum of the cell suspension of each pigmented colony was taken to detect BChl. Thirteen colonies from 17 colored colonies obtained from samples in February showed absorption bands in the 800 to 875 nm region suggesting the presence of BChl *a*. Among the samples collected in July, BChl *a* was detected in 15 out of 51 colored colonies tested. Our results indicate the high abundance of BChl *a*-containing bacteria within cultivable heterotrophic bacteria in the biofilms, *i.e.*, in the winter samples cultivated on 1/10 PEA, all eight colored colonies contained BChl and 80% of the colonies were colored colonies (1.2×10^5^ CFU cm^−2^ of colored colonies in 1.5×10^5^ CFU cm^−2^ of all colonies); in the summer samples cultivated on 1/100 PEA, BChl was detected in four out of the five colored colonies and colored colonies (2.1×10^4^ CFU cm^−2^) accounted for 34% of all colonies (6.2×10^4^ CFU cm^−2^).

In further analyses, the 28 isolates were classified by comparisons of the partial 16S rRNA gene sequences obtained using the sequence primer 27F (600 bp, cut-off value 99.6% identity) and the following 14 strains were selected based on 16S rRNA sequences as representative strains S08, W09, W32, W19, S19, W17, S16, S10, W45, S12, S20, W22, W14, and W35 (Table S1). A total of 1,409 positions in the 16S rRNA gene sequences of the 14 isolates were elucidated and these isolates were divided into 8 OTUs sharing 96% sequence identity (OTU1, S08; OTU2, W09, W32, W19; OTU3, S19, W17; OTU4, S16; OTU5, S10; OTU6, W45; OTU7, S12, S20, W22, W14; OTU8, W35).

### Growth under anaerobic light conditions and aerobic dark conditions

**Fig. 1:**
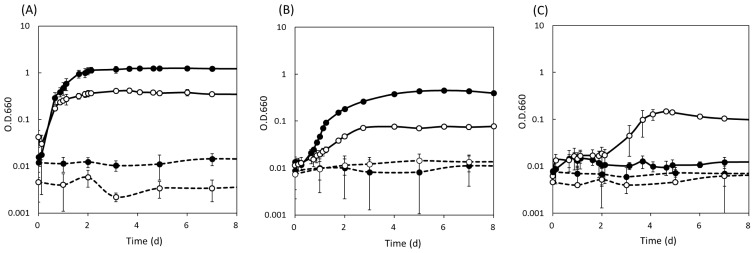
Growth curves of isolates W35 (A), S12 (B), and W32 (C). Cells were cultivated aerobically in the dark (continuous line) and anaerobically in the light (dashed line) using PE medium (closed circle) and 1/5 PE medium (open circle). The error bar shows the standard deviation.

The growth properties of the representative isolates were examined in liquid media (Fig. 1 and S1). None of the isolates grew under anaerobic light conditions. The growth properties of the isolates under aerobic dark conditions were divided into three types: 1) an approximately equal growth rate in PE and 1/5 PE media, as shown in strains W35 (Fig. 1A), W09, W17, S16, and S10 (Fig. S1A to D), 2) a higher growth rate in PE medium than in 1/5 PE medium, as shown in strains S12 (Fig. 1B), S08, and W45 (Fig. S1E and F), and 3) growth in 1/5 PE medium, but no growth in PE medium, as shown in strains W32 (Fig. 1C) and W19 (Fig. S1G).

### Phylogenetic analysis of isolates

**Fig. 2:**
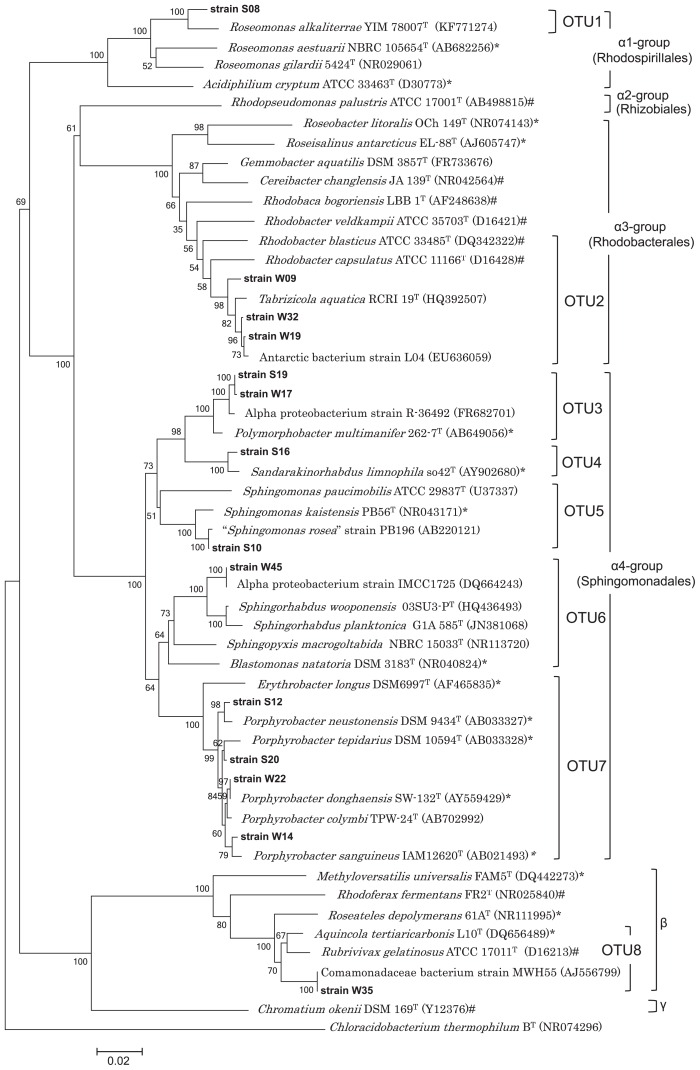
Neighbor-joining phylogenetic tree based on 16S rRNA gene sequences (1,409 positions). The 16S rRNA gene sequences of strains isolated in this study are indicated in bold by the “strain W” and “strain S” prefix in the case of winter and summer isolates, respectively. Sequences from the database are represented with their respective accession numbers behind species and strain names in parentheses. Among these, bacteria known to be AAP bacteria were marked with an asterisk (*) beside the accession numbers. Bacteria known to be anaerobic anoxygenic photosynthetic bacteria (purple photosynthetic bacteria) were marked with a hash (#) beside the accession numbers. α1-group, α2-group, α3-group, and α4-group shown in the right of the tree indicate groups (orders) of the class *Alphaproteobacteria*. β and γ indicate the classes *Betaproteobacteria* and *Gammaproteobacteria*, respectively. The scale bar represents the number of substitutions in each site. Bootstrap values at branch points are expressed as percentages of 500 resamplings. *Chloracidobacterium thermophilum* strain B^T^ (NR074296) was used as an outgroup to root the tree.

Fig. 2 shows a neighbor-joining tree based on 16S rRNA gene sequences from 14 isolates obtained in this study together with those from the database. A phylogenetic tree using the maximum-likelihood method showed a tree topology that was roughly consistent with that in Fig. 2 (data not shown). Eight OTUs were distributed to the orders *Rhodospirillales*, *Rhodobacterales*, and *Sphingomonadales* of the class *Alphaproteobacteria* and the class *Betaproteobacteria*.

The OTU1 isolate S08, belonging to the order *Rhodospirillales*, clustered with *Roseomonas alkaliterrae* (5) as its nearest phylogenetic neighbor. Members of the genus *Roseomonas* are known to produce a pale pink pigment, but only one species of the genus, *R. aestuarii*, has been reported to be a BChl producer (48, 51). Strain S08 (OTU1) is distantly related to the type strain of *R. aestuarii* at a 16S rRNA gene sequence similarity level of 93.4%. OTU2 in the order *Rhodobacterales* contained several winter isolates, which were clustered in a clade with the non-phototrophic bacterium, *Tabrizicola aquatica* (46). Five OTUs, OTUs 3 to 7, belonged to the order *Sphingomonadales*. The OTU3 isolates formed a cluster with *Polymorphobacter multimanifer* as their nearest phylogenetic neighbor and strain S16 (OTU4) and *Sandarakinorhabdus limnophila* as their sister group. *P. multimanifer* (7, 17) and *S. limnophila* (9) are BChl *a*-containing, obligately aerobic bacteria. Strain S10 (OTU5) was found in the cluster of the genus *Sphingomonas*, but the closest relative “*Sphingomonas rosea*” has not yet been reported to contain BChl (41). Bacterial strains related to the genus *Sphingomonas* have also been isolated from river biofilms (28). Strain W45 (OTU6) was closely related to the aerobic heterotrophic bacterium alphaproteobacterium IMCC1725 isolated from a eutrophic pond (40). A large number of isolates obtained from winter and summer samples in this study were clustered in OTU7. All of the isolates in OTU7 were closely related to AAP bacterial species of the genus *Porphyrobacter*. Strain W35 in OTU8 was an isolate belonging to the class *Betaproteobacteria*. The closest relative to strain W35 was the Comamonadaceae bacterium MWH55, isolated from freshwater (accession no. AJ556799).

### BChl contents

Table 2 shows the BChl contents of the isolates grown in PE and 1/5 PE media. Its contents in most of the strains tested were reduced when the isolates were cultivated in non-diluted PE medium, *e.g.*, its content in strain W45 cultivated in PE medium was 20% of that in 1/5 PE medium. BChl contents markedly varied among strains; its lowest content was 0.13 nmol mg^−1^ of dry cell weight in strain S10, whereas its highest was 3.58 nmol mg^−1^ in strain S16 when cultivated in 1/5 PE medium. Previous studies reported that the BChl contents of AAP bacteria were lower than those of phototrophic purple non-sulfur bacteria (16, 39). However, its content in strain S16 was similar to that in *R. sphaeroides* 2.4.1^T^ (ATCC 17023^T^) cultivated photo-anaerobically in 1/5 PE medium.

### Light-harvesting proteins inferred from absorption spectra

The absorption spectra of the isolates after cell disruption were analyzed to characterize light-harvesting proteins. All the isolates listed in Table S1 showed an absorption peak at approximately 870 nm, indicating the presence of light-harvesting 1 complex (LH1) (Table S1 and Fig. S2). Some strains showed the typical absorption of the reaction center (RC)-LH1 complex with a large peak at approximately 870 nm and a small peak at approximately 800 nm (S08, W09, W32, and S12) (Table S1 and Fig. S2A). These strains may lack the light-harvesting 2 complex (LH2). In contrast, strains S19, W17, and S16 may also have LH2, corresponding to the additional absorption peak at approximately 820–850 nm (Table S1 and Fig. S2B). Higher absorption at approximately 800–810 nm than at 870 nm indicated that strain W19 also has LH2 because the absorption of RC-LH1 at approximately 800–810 nm is known to be about 1/15 of that at approximately 870 nm (Fig. S2C) (39).

## Discussion

We investigated the diversity of AAP bacteria in biofilms on submerged river stones using culture-dependent analyses. Phylogenetically diverse AAP bacteria were successfully isolated and their physiological properties were examined.

Previously identified AAP bacteria from various environments are phylogenetically distributed into the classes *Alphaproteobacteria* (*Rhodospirillales*, *Rhizobiales*, *Rhodobacterales*, and *Sphingomonadales*), *Betaproteobacteria*, and *Gammaproteobacteria* (56). The 28 strains isolated in the present study represented the broad diversity of AAP bacteria with representatives from *Alphaproteobacteria* in the order *Rhodospirillales*, *Rhodobacterales*, and *Sphingomonadales* as well as those from *Betaproteobacteria* (Fig. 2) among samples collected from 900 cm^2^ of stone surfaces (150 cm^2^ each from 6 stones) within a 10-m^2^ area. The heterogeneity of microenvironments in epilithic biofilms may allow their co-existence at a small area. Environmental heterogeneity may result in the observed physiological diversity, *i.e.*, preferences for nutrient concentrations (Figs. 1 and S1), BChl contents (Table 2), and *in vivo* absorption spectra (Table S1 and Fig. S2).

Two isolates W19 and W32, belonging to OTU2, grew in low nutrient medium, but not in high nutrient medium (Fig. 1C and S1G). This result suggests that oligotrophic environments were present in the biofilm and these isolates grew in these environments. BChl contents markedly differed among strains and the contents of most isolates were affected by nutrient concentrations (Table 2), as previously reported for other AAP bacteria (14, 39, 42). The availability of light and nutrients may differ in their habitats. Absorption spectra differed among the strains; some isolates had additional absorption bands in the infra-red region, indicating the presence of a peripheral light harvesting complex, while others only had RC-LH1 (Table S1 and Fig. S2), as previously reported for other AAP bacteria (39). Strains with LH2 use light more efficiently; therefore, they may live in the low light intensity environments in biofilms.

Twenty-eight isolates were classified into eight OTUs in the classes *Alphaproteobacteria* and *Betaproteobacteria*. Seven isolates belonging to OTUs 1, 3, 6, or 8 (Fig. 2 and Table S1) likely represent novel species; their 16S rRNA gene sequence identities to established species were 98.0% or less. In contrast, the isolates in OTUs 2, 4, 5, and 7 in the class *Alphaproteobacteria* were closely related to established species. OTUs 4 (1 isolate) and 7 (14 isolates) (Fig. 2 and Table S1) were related to AAP bacteria, which have been isolated from freshwater environments such as lakes and ponds (6, 9). The isolates in OTUs 2 (5 isolates) and 5 (1 isolate) have close relatives, which were not reported to produce BChl or have photosynthetic genes (Fig. 2 and Table S1).

OTU2 clustered phylogenetically apart from established AAP bacteria *e.g.*, *Roseobacter litoralis* and *Roseisalinus antarcticus* in the order *Rhodobacterales* (Fig. 2). These AAP bacteria were isolated from marine and saline environments (56). Our new isolates in OTU2 were the first freshwater AAP bacteria in the order *Rhodobacterales*.

OTU2 contains our 5 isolates as well as *T. aquatica* and some *Rhodobacter* species (Table S1 and Fig. 2). *T. aquatica* was described as a non-phototrophic bacterium devoid of *puf* genes and BChl (46). Our isolates in OTU2 showed a 16S rRNA gene sequence identity of 99.2% to *T. aquatica*. The sequence identities of the OTU2 isolates to the closest relative in the genus *Rhodobacter*, *R. blasticus* were 95.7–96.5%. Members of the genus *Rhodobacter* are known to grow photosynthetically under anaerobic conditions. As shown in a phylogenetic tree focusing on *Rhodobacter* and its relatives (Fig. S3), the large clade comprising *Rhodobacter* species includes several phylogenetic groups as partly suggested by Hiraishi and Ueda (12). Our isolates belonged to a phylogenetic group together with a non-phototroph, *T. aquatica*, and were related to *R. blasticus* (Fig. S3). OTU2 isolates may be an intermediate model of chemotrophs and anaerobic phototrophs.

Strain S10 (OTU5) was closely related to “*S. rosea*” with 99.9% 16S rRNA sequence similarity, which has not been reported to produce BChl or have photosynthetic genes (41). Only some of the species in the genus *Sphingomonas* are known to contain BChl or photosynthetic genes (21, 53). Strain W45 (OTU6) was related to *Sphingorhabdus wooponensis*. No member in the genus *Sphingorhabdus*, including *S. wooponensis*, has been reported to contain photosynthetic pigments or genes (2, 19, 20, 34, 36, 54). These results suggest that phototrophy is no longer useful as a diagnostic phenotypic trait for the classification of organisms at the genus level.

Strain W35 in OTU8 was an isolate in the class *Betaproteobacteria* (Fig. 2). Only two species, *Roseateles depolymerans* (42) and *Aquincola tertiaricarbonis* (27, 35) have been described as AAP bacteria in *Betaproteobacteria*. Other aerobic *Betaproteobacteria* such as *Methyloversatilis unversalis* and *Limnohabitans* sp. were found to have *puf* genes in their genome sequences (22, 58).

Bacteria that have been described as being non-phototrophic and having closely related photosynthetic bacteria including AAP bacteria are assumed to have lost the ability to perform photosynthesis rather recently during their evolution (52). However, it is possible that “non-phototrophic bacteria” actually contain photosynthesis genes and have the ability to produce BChl under some conditions. *Elioraea tepidiphila*, which has been described as a non-phototrophic bacterium, was shown to have *pufLM* gene sequences in its genome (1). *A. tertiaricarbonis* was also initially described as a non-phototrophic bacterium that does not produce BChl, but was subsequently found to produce BChl under different culture conditions (27, 35). These examples suggest that some bacteria described as non-phototrophic may have photosynthesis genes and produce a photosynthetic apparatus under some conditions. We attempted to detect *pufM* gene sequences in our isolates using previously described methods (31, 33), but failed in PCR amplification from most isolates, except for some strains in the order *Sphingomonadales* (data not shown).

It is noteworthy that colonies of AAP bacteria were abundantly observed in plate cultures of biofilm samples. AAP bacteria were frequently isolated from the plate culture of 1/10 PEA or 1/100 PEA medium (Table 1). These isolates were not difficult to cultivate or slowly growing. Traditional cultivation is an effective approach to identify the cultivable diversity of AAP bacteria in river biofilms. A more sensitive detection method for BChl, *e.g.*, a spectrochromatographic method (13) may result in the identification of more BChl-containing isolates.

## Conclusion

This study showed the presence of phylogenetically and physiologically diverse AAP bacteria in river biofilms. Some of the isolated strains were novel lineages. Our previous findings obtained using the culture-independent method suggested that river epilithic biofilms are rich in novel AAP bacteria (15). This culture study confirmed these findings. These diverse AAP bacteria may contribute to material cycling in rivers due to their metabolic potential with the help of energy production through photosynthesis. To the best of our knowledge, this is the first study to demonstrate the phylogenetic and physiological diversities of AAP bacteria in river biofilms.

## Supplementary Information



## Figures and Tables

**Table 1:** Summary of isolation steps from two samples collected in February and July 2012

Media	February 2012				July 2012			
	
	CFU		No. of colonies		CFU		No. of colonies	
			
	Total	Colored	Colored*	BChl**	Total	Colored	Colored*	BChl**
PEA	n.t.	n.t.	n.t.	n.t.	7.6×10^4^	6.0×10^4^	10	2
1/10PEA	1.5×10^5^	1.2×10^5^	8	8	8.4×10^4^	3.4×10^4^	12	6
1/10PEG	1.0×10^5^	2.0×10^4^	2	1	2.0×10^4^	2.9×10^3^	2	0
1/100PEA	3.1×10^4^	6.7×10^3^	2	2	6.2×10^4^	2.1×10^4^	5	4
1/100PEG	2.0×10^4^	6.7×10^3^	5	2	4.0×10^4^	1.5×10^4^	8	2
NA	n.t.	n.t.	n.t.	n.t.	3.3×10^4^	2.9×10^4^	1	0
1/6NA	n.t.	n.t.	n.t.	n.t.	4.4×10^4^	2.7×10^4^	7	0
1/60NA	n.t.	n.t.	n.t.	n.t.	6.1×10^4^	2.4×10^4^	6	1

Media; PEA, PEG, PE medium supplemented with agar or gellan gum, respectively; 1/10, 1/100, 10, or 100 times diluted version of the media; NA, nutrient agar; 1/6NA, 1/60NA, 6, or 60 times diluted NA supplemented with agar up to 1.5% CFU; Total, CFU (cm^−2^ biofilm) calculated by counting the total number of colonies; Colored, CFU (cm^−2^ biofilm) calculated by counting the number of red-, pink-, yellow-, orange- or brown-colored colonies.

n.t., Not tested

* Number of colored colonies tested to have bacteriochlorophyll

** Number of bacteriochrolophyll-containing colonies in colored colonies tested

**Table 2:** Bacteriochlorophyll contents of isolates and *Rhodobacter sphaeroides* strain ATCC 17023^T^

OTU	Strain	BChl content±S.D. (nmol mg^−1^ of dry cell weight)	

		1/5 PE medium	PE medium
1	S08	0.26±0.04	0.44±0.05
2	W09	0.61±0.26	0.29±0.18
3	S19	2.23±1.88	0.95±0.21
4	S16	3.58±2.07	2.90±1.22
5	S10	0.13±0.02	0.00±0.00
6	W45	0.71±0.64	0.15±0.09
7	S12	1.76±0.53	1.53±0.13
8	W35	0.33±0.25	0.07±0.03

	R.sph aerobic	0.08±0.03	0.04±0.03
	R.sph anaerobic	4.12±0.21	10.30±0.10

Each isolate was cultivated aerobically in the dark in liquid medium 1/5 PE or PE. Bacteriochlorophyll was extracted in acetone:methanol.

BChl content: Average value of bacteriochlorophyll content per mg dry cell weight. S.D.: Standard deviation of three cultivations. R.sph aerobic: *R. sphaeroides* strain ATCC 17023^T^ cultivated aerobically in the dark. R.sph anaerobic: *R. sphaeroides* strain ATCC 17023^T^ cultivated anaerobically in the light.
