# Supplementary material for: Phylogenetically Diverse Aerobic Anoxygenic Phototrophic Bacteria Isolated from Epilithic Biofilms in Tama River, Japan
Source: Microbes Environ. 2016 Jul 23;31(3):299–306. doi: 10.1264/jsme2.ME15209 (PMC5017807; doi:10.1264/jsme2.ME15209)
Supplement: Supplementary file 1 [file 31_299_s1.pdf]

Supplemental Materials

Table S1. A list of 14 representative isolates in this study

OTU	Strain	Medium	Related isolates	Absorption of BChl			Closest relative (Accession no.)	Identity (%)
1	S08	1	-	798		866	*Roseomonas alkaliterrae* YIM78007^{T} (KF771274)	97.4
2	W09	3	-	803		871	*Tabrizicola aquatica* RCRI19^{T} (HQ392507)	98.4
2	W19	1	-	804		866	*Tabrizicola aquatica* RCRI19^{T} (HQ392507)	98.8
2	W32	3	W07 W40	802		868	*Tabrizicola aquatica* RCRI19^{T} (HQ392507)	99.2
3	S19	1	-	801	838	867	*Polymorphobacter multimanifer* 267-2^{T}* (AB649056)	98.0
3	W17	1	W04	804	822	868	*Polymorphobacter multimanifer* 267-2^{T}* (AB649056)	98.0
4	S16	3	-	800	852	872	*Sandarakinorhabdus limnophila* so42^{T}* (AY902680)	98.6
5	S10	6	-			874	"*Sphingomonas rosea*" strain PB196 (AB220121)	99.9
6	W45	1	W02			864	*Sphingopyxis wooponensis* 03SU3-P^{T} (HQ436493)	97.8
7	S12	5	S07	800		872	*Porphyrobacter neustonensis* DSM 9434^{T}* (AB033327)	99.2
7	S20	5	S02 S03 S05 S14			866	*Porphyrobacter colymbi* TPW-24^{T} (AB702992)	99.5
7	W22	2	S04			868	*Porphyrobacter donghaensis* SW-132^{T} * (AY559428)	99.9
7	W14	1	W34 S01 S06 S13			860	*Porphyrobacter colymbi* TPW-24^{T} (AB702992)	99.4
8	W35	4	-			871	*Aquincola tertiaricarbonis* L10^{T}* (DQ656489)	97.3

Medium : medium the strain was isolated from; 1, 1/10PEA; 2, 1/10PEG; 3, 1/100PEA; 4, 1/100PEG; 5, PEA; 6, 1/60NA

Absorption of BChl : *In vivo* absorption peaks of bacteriochlorophyll (nm).

* AAP bacteria.

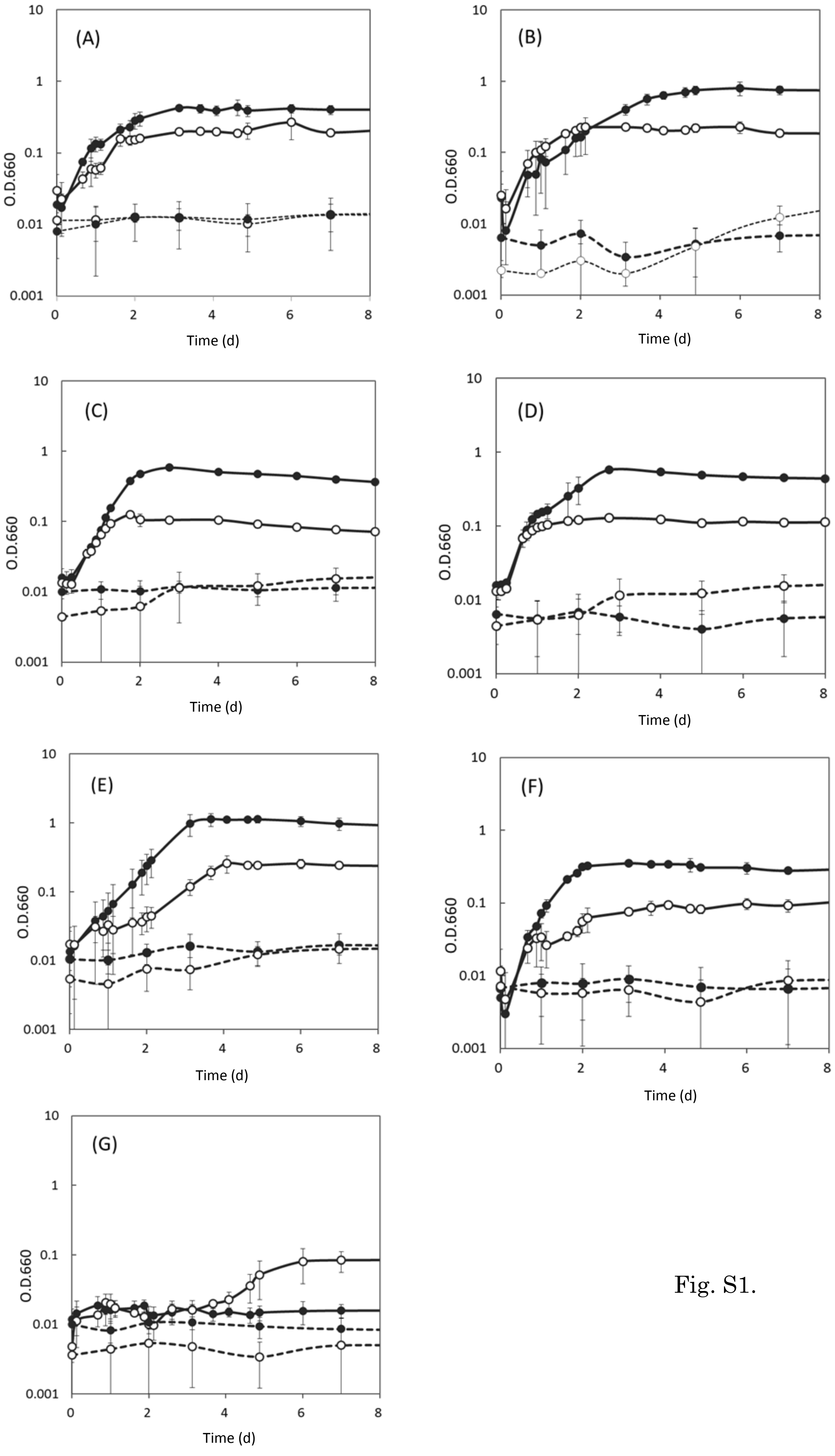

Fig. S1.

Fig. S1. Growth curves of isolates W09 (A), W17 (B), S16 (C), S10 (D), S08 (E), W45 (F), and W19 (G). Cells were cultivated aerobically in the dark (continuous line) and anaerobically in the light (dashed line) using PE medium (closed circle) and 1/5 PE medium (open circle). The error bar shows the standard deviation.

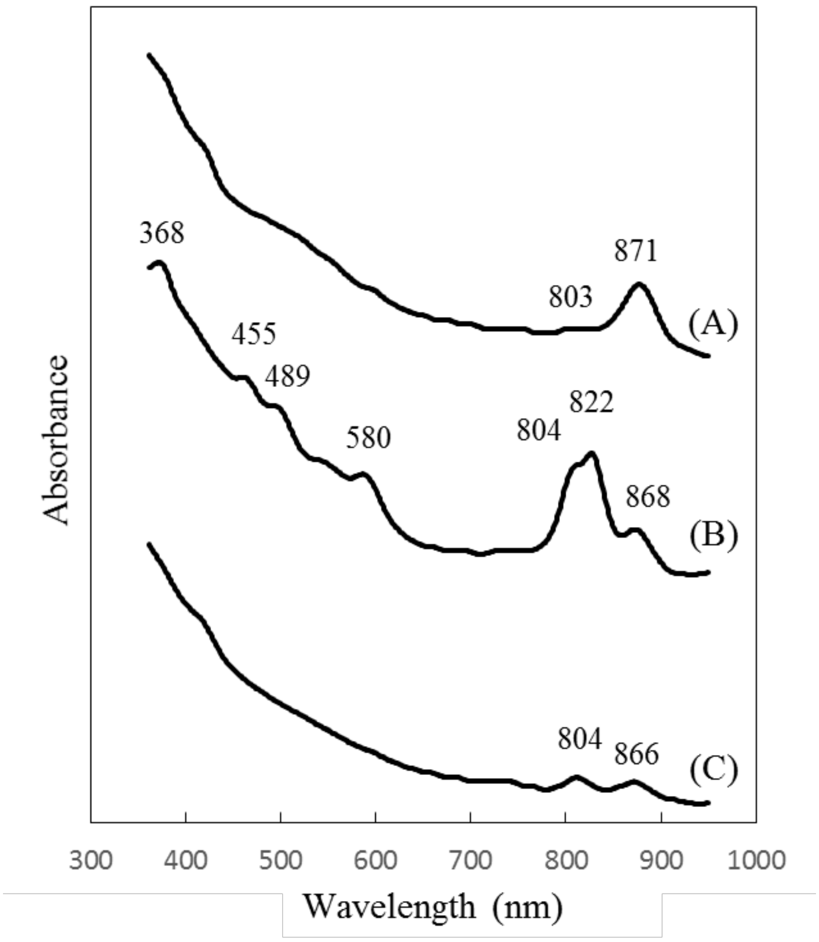

Fig. S2. *In vivo* absorption spectra of strains W09 (A), W17 (B), and W19 (C). Cells were cultivated aerobically in the dark for 7 d using 1/5 PE medium. Harvested cells were washed and disrupted in 10mM Tris-HCl buffer pH8.0. Cell debris were removed by centrifugation.

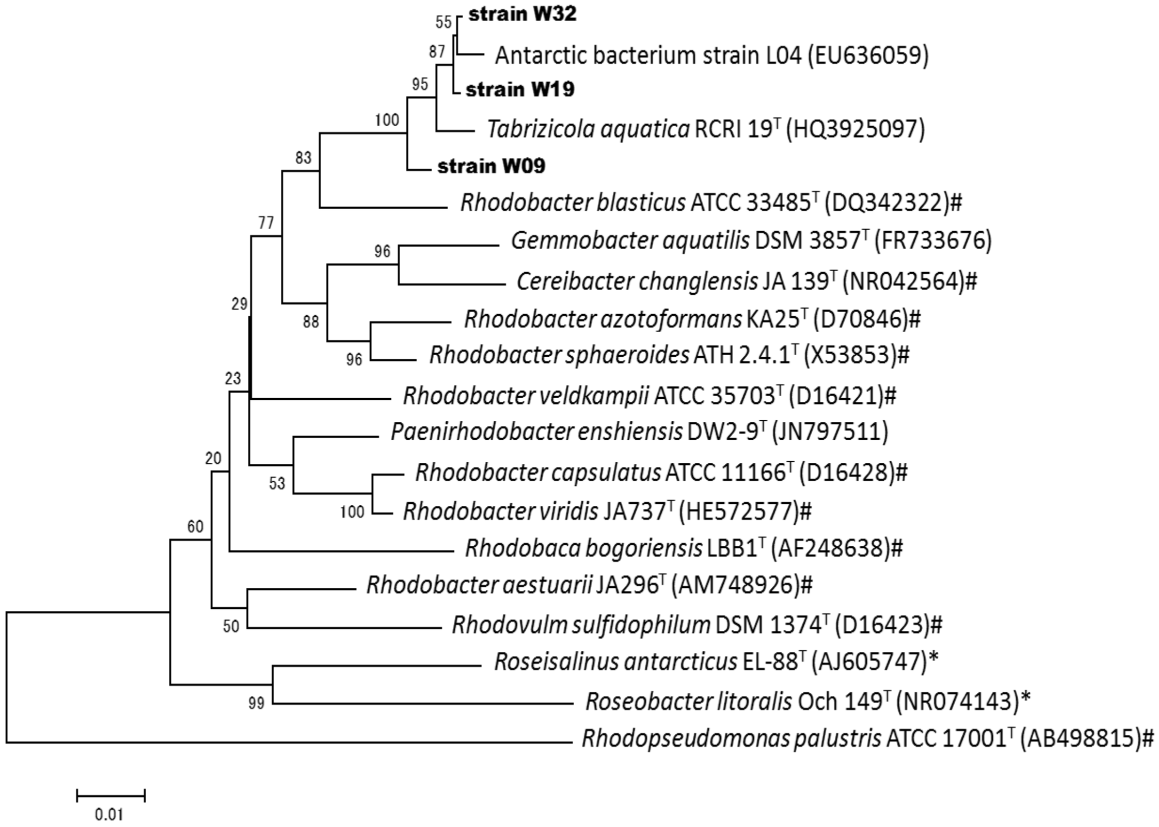

Fig. S3. Neighbor-joining phylogenetic tree of *Rhodobacterales* species based on 16S rRNA gene sequences (1,409 positions). The 16S rRNA gene sequences of strains isolated in this study are indicated in bold by the “strain W” prefix. Sequences from the database are represented with their respective accession numbers behind species and strain names in parentheses. Among these, bacteria known to be AAP bacteria were marked with an asterisk (*) beside the accession numbers. Bacteria known to be anaerobic anoxygenic photosynthetic bacteria (purple photosynthetic bacteria) were marked with a hash (#) beside the accession numbers. The scale bar represents the number of substitutions in each site. Bootstrap values at branch points are expressed as percentages of 500 resamplings. *Rhodopseudomonas palustris* strain ATCC 17001^{T} (AB498815) was used as an outgroup to root the tree.
